# The impact of the COVID-19 pandemic on firms: a survey in Guangdong Province, China

**DOI:** 10.1186/s41256-020-00166-z

**Published:** 2020-09-02

**Authors:** Peng Zou, Di Huo, Meng Li

**Affiliations:** School of Management, Harbin Institute of Technology, No. 13 Fayuan District, Harbin, 150001 China

**Keywords:** COVID-19 pandemic, Economic impact, Public policy, Health risks

## Abstract

**Background:**

The COVID-19 pandemic has affected all sectors of the world economy and society. To understand the impact of the pandemic on firms in China and suggest public policies to deal with the negative effects, we investigated firms in Guangdong Province.

**Methods:**

The survey sample included 524 firms in 15 cities in Guangdong Province. We chose these firms from the list published by the government, considering the industrial characteristics of Guangdong province and firm size. The questionnaire was developed based on previous studies carried out by UNDP. It comprised four categories with a total of 17 questions. The executives of firms were contacted by telephone or WeChat, and were invited to answer self-administered questionnaires through an online survey platform. The data was analyzed by SPSS.

**Results:**

The following key findings were revealed through the survey: (1) 48.7% of firms maintained stability, and 35.1% experienced a halt in operation or faced closure; (2) Nearly 70–90% already exploit online commerce or are willing to do so, and also remote office work, and digital operations. (3) 46% believe that they will certainly incur losses for 2020, and 83.5% expect the city’s GDP to decrease.

**Conclusions:**

Firms in Guangdong Province have faced great challenges in the epidemic. Their production and operation activities have been limited, and they are facing significant risks. It is necessary to implement policies that would profoundly lower production costs for firms, help them survive this difficult period, and gradually return to normal business.

## Background

The current COVID-19 is a rapidly evolving global challenge and like any pandemic, it weakens health systems, costs lives, and also poses great risks to the global economy and security [1–4]. According to data from World Health Organization (WHO) and Johns Hopkins University, as end- of June 2020, there have been ten million cases around the world, causing nearly 500,000 deaths in around 215 countries (https://www.arcgis.com/apps/opsdashboard/index.html). The COVID-19 pandemic is a public health emergency and is likely to continue to cause serious public health damage including major infectious diseases, mass unexplained diseases, major food poisoning and other serious public health issues [5]. Global economic growth is expected to decrease continually [6, 7] and according to the an OECD forecast, the global GDP growth rate will drop to 2.4% in 2020 [3]. The risk of uncertainty and global recession has increased greatly [8, 9],^1^ due to disruptions in the supply chain, shrinking demand for consumption and investment, significant weakening of economic activities, and damaged market confidence. These factors are severely testing the resilience of many economies, the level of governance, and the effectiveness of international cooperation.

The impact of the epidemic on China’s economy is significant [10–14]. In the first two months of 2020, China’s added value in terms of size, investment, and consumption fell by 13.5, 24.5, and 20.5% year-on-year, and the unemployment rate reached 6.2%, for the first quarter of 2020, China’s GDP growth rate fell by 6.8%, according to the data released by the National Bureau of Statistics of China on April 17 [15].

As the basic unit of the national economy, the operation of firms is key to the development of the national macro-economy. Therefore, it is necessary to understand the status of firms, their coping strategies, and the need for government policies to respond to the impact of the COVID-19 pandemic. Researchers have begun to analyze firms’ marketing innovation strategies and strategic responses to the crisis during the pandemic [16–18]. In addition, some industries have also been analyzed in order to propose more targeted countermeasures for the pandemic [19]. However, the conclusions of these studies have not been empirically tested.

In this context, in order to explore the impact of the COVID-19 pandemic on firms in China and to suggest how public policies might be formulated to deal with the crisis, we investigated firms in Guangdong Province (the province with the highest GDP in China). The purpose of this research was to investigate 1) the impact of the pandemic on firms, 2) how they responded, and 3) and their expectations for the future. Some supporting policies are recommended.

## Methods

### Study setting and design

The research protocol was approved by the Institutional Review Committee of the School of Management, Harbin Institute of Technology, with approval number 2020–01. A survey was designed to be carried out in Guangdong Province.

The minimum sample size was calculated based on the formula below:

1 $$ \mathrm{Sample}\kern0.17em \mathrm{Size}={z}^2{\sigma}^2/{E}^2 $$

where E = 0.05 (margin of error); z = 1.96 (confidence level); and σ = 0.5.

Based on the above parameters, the estimated minimum sample size was 384. However, in order to improve the reliability of the data, the sample was increased to over 500. The survey sample included 524 firms distributing in 15 cities including Guangzhou, Shenzhen, Dongguan, and Foshan (There are 21 cities in Guangdong Province and the GDP of the 15 cities included in our sample represent more than 90% of the total GDP of Guangdong Province). These firms were selected from a list of firms published by the government, considering the industrial characteristics of Guangdong province and the size of firms.

### Questionnaire development

The questionnaire was developed based on previous studies carried out by United Nations Development Program (UNDP) in China [11]. It was piloted among 10 CEOs and firm owners. The questionnaire was further revised based on their feedback along with from two senior experts [20]. The final questionnaire included 17 questions distributed in four categories: demographic characteristics (3 questions); the impact of the pandemic on firms (6 questions); firms action (5 questions); and firm perceptions (3 questions) (Appendix 1).

### Data collection

The survey was carried out from 10 April 2020 to 25 April 2020. Three researchers contacted the executives of firms via telephone or WeChat, and invited them to answer the self-administered questionnaires on “WJX”, an online survey platform (https://www.wjx.cn/). Participation in the survey was fully voluntary and written consent was obtained from each participant. The objectives of the study, confidentiality of individual information, and other ethical considerations mentioned in the survey guidelines were explained to the participants prior to data collection. They were asked to answer as many of the questions as they could. However, if they were not sure about the answer, they could simply leave it blank. Altogether, 553 anonymous questionnaires were collected.

### Data processing and analysis

The data was entered into Excel for data documentation. SPSS was applied to further analyze the data. A total of 29 participants were excluded from the sample because they answered less than 70% questions, and missing some key answer s. Descriptive statistics of demographic characteristics of the sample and each item of questions were employed to summarize the data.

## Results

### Demographic characteristics of the sample

The effective response rate of the sample was 94.8% (524/553). The industries in the effective response investigation were Information technology (IT) (18%), manufacturing (34.6%), finance (12.4%), real estate (6.7%), and service and commercial industry (23.7%). Other industries accounted for 4.8%. This distribution is in line with the Guangdong’s industrial characteristics. The size of the surveyed firms was relatively balanced. A total of 141 firms accounted for 26.9% of all the firms and have 50 employees or fewer, 162 firms (30.1%, 50–500 employees, 129 firms (24.4%, 501–5000 employees), and 92 firms 17.6%, 5000 employees or more). Table 1 shows the demographic characteristics of the firms by number and percentage, including industry, size, and location.

**Table 1:** Overview of the sample of firms

	Field of business	Number of Firms	Percentage
Industry	Sub-industry		
IT	/ Software and hardware services / E-commerce / Internet operations	**94**	**17.94%**
Manufacture	Main Manufacture	88	16.79%
	Electronic technologies / Semiconductors / Integrated circuits	14	2.67%
	Clothing / Textiles / Leather	7	1.34%
	Aerospace / Aviation / Energy / Chemical	10	1.91%
	Machinery / Equipment / Heavy industry	10	1.91%
	Electric appliances	5	0.95%
	Furniture / Crafts / Toys	6	1.15%
	FMCG (food / beverage / cosmetics)	12	2.29%
	Automobiles and spare parts	9	1.72%
	Medical / Nursing / Health / Sanitation	8	1.53%
	Instrumentation / Industrial Automation	5	0.95%
	Pharmaceutical / Bioengineering / Medical Equipment	15	2.86%
		**181**	**34.6%**
Service and Commercial	Publishing / Printing / Packaging	5	0.95%
	Advertising / PR / Media / Art	4	0.76%
	Law	1	0.19%
	Accounting / Auditing	1	0.19%
	Traffic / Transportation / Logistics	11	2.10%
	Education / Training / Scientific research / Colleges	15	2.86%
	Trading / Import & Export	13	2.48%
	Wholesale / Retail	13	2.48%
	Communication / Telecommunications / Network equipment	21	4.01%
	Property Management / Commercial Centers	8	1.53%
	Agency / Consulting / Headhunting / Certification	12	2.29%
		**124**	**23.7%**
Finance	Bank / Insurance / Securities / Investment Bank / Risk Fund	**65**	**12.40%**
Real estate and Architecture	Real estate development / Architectural engineering / Design	**35**	**6.68%**
Other industry		**25**	**4.77%**
	Employees		
	50 and below	141	26.91%
	51–100	54	10.31%
	101–300	71	13.55%
	301–500	37	7.06%
	501–1000	42	8.02%
	1001–4999	87	16.60%
	5000 and above	92	17.56%
	Total	524	100%

### The impact of the pandemic on firms

Although half of the firms maintained their operations and overall stability, many experienced a halt in operations or faced closure for various reasons such as shortages of materials and stock (Q1 and Q2).

Firms are suffering from at least one of the following pressures: employees’ salary and social insurance, rent, loss of orders, payment of accounts payable, and loan repayment (Q3). For firms with fewer than 50 employees, rent payments seem to be the key pact.

Most firms barely maintained production, facing a shortage of materials or lack of supply (Q4). A total of 22.9% of firms had orders from domestic customers cancelled, and 63.9% had overseas customers who cancelled orders or failed to send supplies on time.

**Fig. 1:**
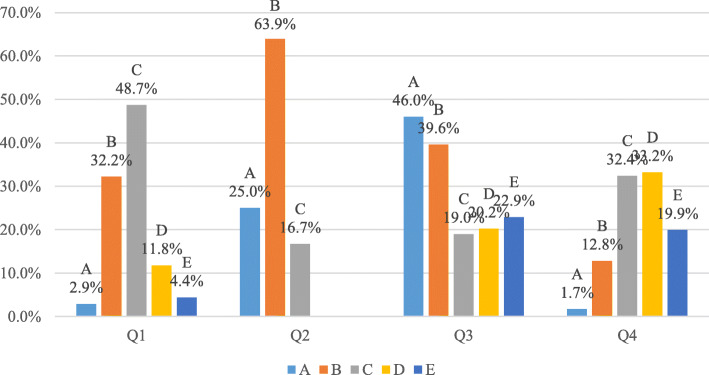
Descriptive statistics of Q1--Q4. Note: Appendix 1 contains details of the questions and options

Descriptive statistics can be found in Fig. 1.

### How firms responded to the crisis

Firms recognized the problems and devoted more efforts to R&D and innovation. However, the epidemic has delayed the launch of new products as well as the progress of projects in cooperation with other institutions, especially for the IT industry (Q7). Many firms have faced higher labor costs, which have forced them to consider reducing the number of employees, cutting wages, and postponing recruitment until the effects of the pandemic are over (Q5, Q6 and Q8). Manufacturing has been facing a higher proportion of employment difficulties, and the real estate industry has been shedding the jobs. Both IT and Manufacturing industries have also sought out loans as well as funding from shareholders (Q8).

A high percentage of firms have already moved their operations to online or plan to do so (Q9). Likewise, many firms have already started working remotely and digital operations or plan to do so (Q10 and Q11).

**Fig. 2:**
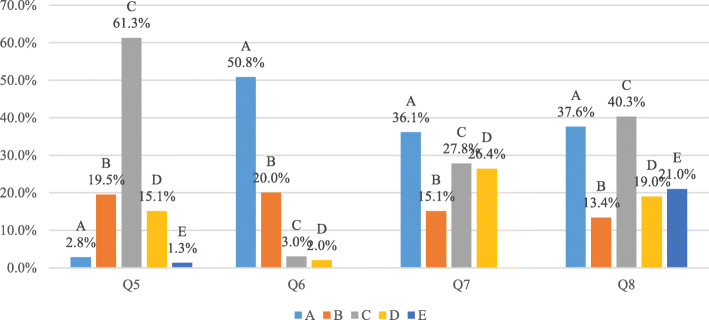
Descriptive statistics of Q5-Q8. Note: Appendix 1 contains details of the questions and options

**Fig. 3:**
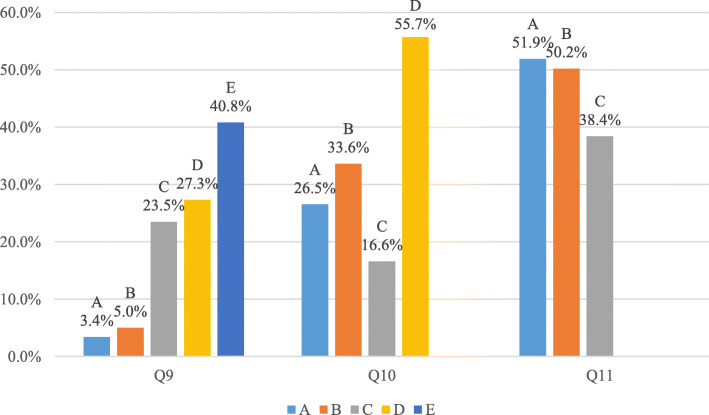
Descriptive statistics of Q9-Q 11. Note: Appendix 1 contains details of the questions and options

Descriptive statistics can be found in Fig. 2 and Fig. 3.

### The expectations of firms

A high percentage of firms believe that they will experience financial losses this year, while a lower percentage think they will be able to make a profit (Q13). However, firms with more than 1000 employees appear to be in a relatively good position to survive, and some believe that they will certainly make a profit and have sufficient cash flows.

More firms expected their local town to experience a decrease in GDP than those who thought that GDP will remain unchanged or even increase (Q14).

Most firms felt that the government needs reduce, exempt, or postpone social insurance, value added tax, income tax and other taxes (Q12). More than half of the firms expect that the government to stimulate consumption. They also need subsidies, for example, for rent, utilities, and post stabilization some firms expect to make a staged flexible salary. Smaller firms need more cash subsidies, while larger firms tend to get policies of extending loan repayment terms and debt forgiveness.

**Fig. 4:**
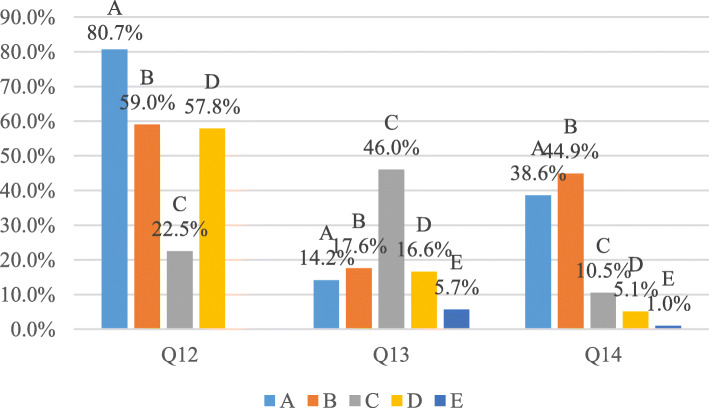
Descriptive statistics of Q12--Q14

Descriptive statistics can be found Fig. 4.

## Discussion

Most of the research on public health emergencies has focused on changes in overall consumption trends, such as consumption behavior, changes in consumer decision-making and consumer psychology [21–24]. These studies show that pandemics reduce economic activity and employment, and inhibit consumption, which is in line with our findings on the impact of the COVID-19 pandemic. However, such studies only analyzed the trend in overall consumption, while we investigated the firms themselves, thereby more comprehensively reflecting the impact of the COVID-19 pandemic on economic activities.

### The impact of the pandemic on firms

Given the pandemic, returning to work was not the same as reaching production capacity. In fact, almost half of the firms were facing material shortages, some of them even said that they had run out of supplies (see answers to Q1 and Q2). In response to Q3 regarding operational pressures, our survey revealed that firms were facing employment and costs. In addition, 22.9% of firms had had orders cancelled from domestic customers. Many firms responded that the market expansion was also affected, due to the impossibility of site visiting and face-to-face meetings with customers.

Moreover, more than half of the firms reported a sharp increase in operating costs such as the cancellation of orders, delayed delivery, and production stagnation. Some of them had to deal with insolvency and compensation for breach of contract. Firms were also under the pressure of employees’ salary, insurances, rent payments, settlement of accounts payable, and loan repayment.

As highlighted by the answers to Q4 (supply of raw materials, spare parts and other production and operation materials), the business owners in the interviews reported that supply chain links - the producing and processing of raw material parts, and logistics etc. were affected by the epidemic situation in various regions, with different degrees of delay in delivery and work stoppages while waiting for materials. The spread of epidemic to outside China affected many export businesses. Firms reported cancellation of orders or delayed receipt of goods from overseas customers.

### How firms responded to the epidemic

According to the answers for Q5, Q6 and Q8, the epidemic also negatively impacted the technological innovation and human resources in some firms. For example, the epidemic has delayed the launch of new products, new recruits, and the progress of projects in cooperation with other firms.

According to Q7, the combination of this pandemic and the impact of the Sino US trade dispute prompted some firms to recognize the problems and to devote more efforts to R & D and innovation.

Q9 and Q10 indicate that, more than half of the firms believe that this pandemic has promoted the establishment of remote offices, remote recruitment, and remote business negotiation models, and the informatization and digitalization of firms have improved their ability to respond to major crises. A few firms also mentioned that the epidemic can eliminate competitors to a certain extent, by triggering the launch of new businesses, and accelerating changes in marketing approaches (such as community channel expansion). For example, the core business of clothing brand PeaceBird was offline retailing in large shopping malls. During the crisis, they transferred employees from offline stores to online sales teams. They successfully completed this channel transformation and survived the crisis by using livestreaming and social media platforms [25].

The epidemic has strengthened the determination of some firms to turn crises into opportunities and invest in new industries. At the same time, it has strengthened consumers’ health awareness and changed their consumption behaviors [26–28]. Some firms reported that this trend has led to new business opportunities and has driven reforms in their marketing strategies. The interviewees indicated that there is a need to accelerate, for example, the development of online education, medical care, and 5G.

Some firms have changed the original store-based marketing mode and integrated channels for interactive marketing. For example, the store is not open, but the sales people conduct live broadcasts, group buying and social marketing, to name a few.

### The expectations of firms

The result of this investigation indicate that rebound consumption will not come. Consumption is mainly determined by demand and the ability to pay [29]. The ability to pay depends on the consumer’s current income and expectations of future income [30]. Since the epidemic has continued, almost half firms believe that there they will definitely incur losses this year.

Q13 asked “To what extent do you expect this pandemic will affect your firm’s development in the first quarter of 2020?” and nearly half of firms predicted that losses will directly affect their employees’ income and even work opportunities. More than 20% of firms have reduced the number of staff to cope with the epidemic.

Moreover, respondents are also not optimistic about the GDP growth of the city where the firm is located (Q14). Among them, 83.5% expected a decreasing trend in their city’s GDP growth, and 38.6% predicted a significant decline. On the contrary, only a few respondents believed that GDP would remain unchanged or anticipated that GDP would increase. Therefore, there is insufficient support for any consumption rebound given expectations for reduced income and spending power.

Unfortunately, the Spring Festival, which took place at the height of this pandemic, would normally stimulate a high level of consumption [31] over a short period which is not protracted throughout the year [32]. In the long run, the epidemic will reduce people’s willingness to consume apart from necessities and immediate needs. In the short term, consumer psychology has not fully returned to normal [33], and establishing a work mode that is compatible with epidemic prevention and control takes time. Therefore, the endogenous power of compensatory and rebound consumption is insufficient.

### Policy implications

On the basis of our survey and considering the firms’ appeal for government support, we believe that the following financial policies would stimulate consumption and help firms to survive.

1. Financial policies supporting production and operation. Firms expect public policies and measures such as the “tax exemption, reduction, postponement, return and compensation”, in order to substantially lower production costs for firms, and help firms survive the difficult period of production and operation, while gradually returning to normal business operations.
2. Investment driving consumption. Investment should play a driving role in promoting consumption. Investment in fields such as public health facilities and health care should be increased to cultivate and expand consumer demand.
3. Measures supporting and facilitating consumption. Holiday tourism products could be launched alongside a paid vacation system. Policies should be targeted to promote tourism and the ‘night economy’.
4. Role of industry organizations in promoting consumption. Industry organizations could release suppressed and frozen consumption by carrying out various activities such as shopping festivals and food festivals to stimulate consumption; and facilitate an environment for promoting online-to-offline integrated consumption, for example through preferential concessions, model innovation, policy stimulation, cooperation between banks and firms, strengthening services, and product innovation.

### Limitation of this study

Our study has several limitations that provide opportunities for future research. First, this study can only be viewed as a preliminary study and more follow-up tracing investigations at different stages need to be conducted in order to monitor the continuing impact of the pandemic and the effectiveness of public policies and firms’ responses. Second, this study focuses on China which was among the first countries to recover from the pandemic, more surveys should be implemented in other countries to explore how COVID-19 has impacted other cultural, social and governing systems. A cross-sector analysis is also needed in order to get more specific suggestions for different industries in different regions.

## Conclusions

While many firms in Guangdong Province have maintained overall stability, others have experienced a halt in their operations or faced closure. Almost all the firms in our survey are willing to transform into online marketing, remote office work and digital operations. Half of the firms believe that there will be a certain loss this year, and a very high percentage of firms expected a decreasing trend in the city’s GDP growth.

There is a need to fully understand the impact of the epidemic on consumption and the difficulty of promoting the recovery of consumption, also in terms of how industry has been affected by this pandemic. Policies need to be introduced to profoundly lower production costs for firms, and help firms survive this difficult period, and gradually return to normal business operations.

## Data Availability

Please contact the corresponding author for data requests.

## Appendix

### Questionnaire

#### Survey on firms affected by the COVID-19 Pandemic

1. To what extent does has production and operations of your firm been affected by this pandemic? (single choice)

**Table:**

Options	
A. Very serious impact, leading to serious difficulties in business operations and bankruptcy	
B. Great impact: operations barely maintained	
C. Small impact, some difficulties in business operations, but overall stability	
D. No significant impact	
E. Positive impact, providing new opportunities for development	

2. What are the reasons for the suspension of production and operations of your firm? (multiple choice, up to 2 items)

**Table:**

Options	
A. Shortage of production materials.	
B. Difficulty in developing market	
C. Impact of measures taken to respond to the pandemic	

3. What are the main operating pressures that your firm is currently facing? (multiple choice, up to 3 items)

**Table:**

Options	
A. Employee salaries, insurances	
B. Rent (Buildings, Equipment)	
C. Repayment of loans	
D. Payment of accounts payable	
E. Cancellation of orders	

4. What is the current situation regarding the supply of raw materials, spare parts and other production and operation materials in your firm? (single choice)

**Table:**

Options	
A. Total disruption of supply	
B. Supply shortage	
C. Supply barely maintains production	
D. Satisfactory supply	
E. Normal supply	

5. Does your company plan to reduce or increase the number of employees? (single choice)

**Table:**

Options	
A. Reduce greatly(30–50%)	
B. Reduce slightly(10–30%)	
C. Remain basically the same	
D. Increase slightly(10–30%)	
E. Increase greatly(30–50%)	

6. How has the pandemic affected recruitment? (multiple choice, up to 3 items)

**Table:**

Options	
A. Increase in labor costs	
B. Unable to find a suitable recruitment channel	
C. Postponement or cancelation the existing recruitment plan	
D Transition to online recruitment	

7. What is the clearest impact of the pandemic on your firm’s technological innovation? (multiple choice, up to 3 items)

**Table:**

Options	
A R&D process may affect the launching process of new product	
B Unable to recruit suitable R&D personnel	
C. Unable to cooperate with other departments to carry out part of R & D work	
D. Determined to invest more in technological innovation after being better aware of the firm’s self-development problems during the pandemic	

8. How are you currently or planning to cope with the cash flow shortage? (multiple choice). WHAT IF THEY DON’T HAVE a CASH FLOW SHORTAGE?

**Table:**

Options	
A. Funding from existing shareholders	
B. Adding new shareholders	
C. Loans	
D. Delaying payment	
E. Cutting pay and jobs	

9. Are you willing to transform to online commerce?

**Table:**

Options	
A. Very unwilling	
B. Unwilling	
C. Reasonably willing	
D. Willing	
E. Very willing	

10. What self-help measures has your firm taken so far? (multiple choice)

**Table:**

Options	
A. Applied for financing	
B. Increased online operations	
C. Cut pay and jobs.	
D. Implemented a remote office (digital office)	

11. What are the potentially positive impacts of the pandemic in your view? (multiple choice, up to 3 items)

**Table:**

Options	
A. Promote the establishment of remote office work	
B. Enhance information and digital construction of firms	
C. Help to better realize firm’s shortcomings and solve existing problems	

12. What policies do you expect the government will put in to place to help your firm overcome the difficulties? (multiple choice, up to 4 items)

**Table:**

Options	
A. Reduce, exempt or postpone value-added tax, income tax, insurance premiums and other taxes	
B. Stimulate consumption	
C. Allow firms to implement a staged flexible salary method	
D. Provide subsidies for rent, utilities, post stabilization etc.	

13. To what extent do you expect this pandemic will affect your firm’s development in the first quarter of 2020?(single choice)

**Table:**

Options	
A. Profits	
B. Balance of income and expenditure	
D. Losses	
E. Serious losses	
A. Bankruptcy	

14. What has been the effect on your city’s economic (GDP) growth in the first quarter of this year?

**Table:**

Options	
A. Reduced significantly	
B. Reduced slightly	
C. Unchanged	
D. Increased slightly	
E. Increased significantly	

15. Where is your firm located?

**Table:**

Options	
Chaozhou	
Dongguan	
Foshan	
Guangzhou	
Huizhou	
Jiangmen	
Maoming	
Shantou	
Shenzhen	
Yangjiang	
Zhanjiang	
Zhongshan	
Zhuhai	

16. What industry does your firm belong to?

**Table:**

Options	
IT / Software and hardware services / E-commerce / Internet operations	
Catering / Entertainment / Tourism / Hotel	
Publishing / Printing / Packaging	
Electronic technology / Semiconductor / Integrated circuit	
Law	
Real estate development / Architectural engineering / Design	
Clothing / Textile / Leather	
Advertising / PR / Media / Art	
Aerospace / Aviation / Energy / Chemical	
Accounting / Auditing	
Machinery / equipment / Heavy industry	
Home appliance industry	
Furniture / Crafts / Toys	
Traffic / Transportation / Logistics	
Education / Training / Scientific research / College	
FMCG (food / beverage / cosmetics)	
Trading / Import & Export	
Agriculture / Fishery / Forestry	
Wholesale / Retail	
Automobile and spare parts	
Communication / Telecommunications operations / Network equipment	
Property Management / Commercial Center	
Medical / Nursing / Health / Sanitation	
Instrumentation / Industrial Automation	
Bank / Insurance / Securities / Investment Bank / Risk Fund	
Pharmaceutical / Bioengineering / Medical Equipment	
Manufacture	
Consulting / Headhunting / Certification	
Others	

17. How many employees are there in your company?

**Table:**

Options	
50 and below	
51–100	
101–300	
301–500	
501–1000	
1001–4999	
5000 and above	

## Abbreviations

COVID-19: Coronavirus disease 2019
OECD: Organization for Economic Co-operation and Development
GDP: Gross Domestic Product
WHO: World Health Organization
IMF: World Health Organization
CEO: Chief Executive Officer
SPSS: Statistical Product and Service Solutions
R&D: Research and Development
UNDP: United Nations Development Program

## Footnotes

1 The International Monetary Fund (IMF) broadly defined the global economic growth rate to fall below 2.5% per year as a global recession.
